# Comparison of Q-value-guided laser-assisted in situ keratomileusis and standard laser in situ keratomileusis for myopia: A meta-analysis

**DOI:** 10.1097/MD.0000000000021563

**Published:** 2020-11-06

**Authors:** Kai-Ping Zhang, Xiang Fang, Yin Zhang, Min Chao

**Affiliations:** Department of Urology, Anhui Provincial Children's Hospital and Children's Hospital of Anhui Medical University, Hefei, Anhui, P. R. China.

**Keywords:** laser in situ keratomileusis, myopia, *Q*-value, meta-analysis

## Abstract

**Background:**

Previous studies examining the safety and efficacy of *Q*-value-guided laser-assisted in situ keratomileusis (LASIK) for treating myopia have yielded inconsistent results. We, therefore, performed a meta-analysis to clarify this issue

**Methods:**

Various databases were conducted up to November 21, 2018. All randomized controlled trials and cohorts that compared *Q*-value-guided LASIK with standard LASIK were selected. Mean differences (MDs) or odds ratios (ORs) with 95% confidence intervals (CIs) were calculated to evaluate the strength of the correlations. Additionally, different subgroup analyses and publication bias tests were performed. Data were extracted including the number of postoperative uncorrected visual acuity (UCVA) of 20/20 or better, postoperative UCVA, preoperative and postoperative *Q*-value, postoperative refractive spherical equivalent (SE), the number of postoperative SE within ±0.5D, higher order aberration (HOA), coma-like aberration and spherical-like aberration.

**Results:**

A total of seventeen studies with 2640 patients and 3,358 eyes were included. It has been shown that postoperative *Q*-value (MD = -0.42; 95% CI: -0.64, -0.21; *P* < .001), HOA (MD = -0.14; 95% CI: -0.23, -0.06; *P* = .001), spherical-like aberration (MD = -0.19; 95% CI: -0.32, -0.06; *P* = .004) rather than postoperative UCVA (MD = 0.04; 95% CI: 0.01, 0.07; *P* = .012) were significantly better in the *Q*-value-guided LASIK than standard LASIK. However, the pooled results revealed that no significant differences were found between the 2 paired groups of postoperative UCVA of 20/20 or better (OR = 1.09; 95% CI: 0.62, 1.92; *P* = .763), preoperative *Q*-value (MD = -0.00; 95% CI: -0.02, 0.02; *P* = .922), postoperative refractive SE (MD = 0.08; 95% CI: -0.09, 0.25; *P* = .336), coma-like aberration (horizontal: MD = -0.00; 95% CI: -0.03, 0.03; *P* = .966; vertical: MD = -0.01; 95% CI: -0.03, 0.01; *P* = .263) and postoperative SE within ±0.5 D (OR = 1.06; 95% CI: 0.48, 2.33; *P* = .886). Likewise, similar results were detected in some corresponding subgroups.

**Conclusion:**

*Q*-value-guided LASIK is a safe, effective and predictable surgical option for treating myopia, especially showing superiority over standard LASIK in postoperative *Q*-value, HOA and spherical-like aberration. However, more detailed studies are required to confirm our conclusions in advanced researches.

## 1 Introduction

Myopia is a common eye disease which increasingly recognized as a significant cause of visual impairment and blindness globally. Recent evidences from epidemiological studies suggested a increasing prevalence of myopia, causing a profound economic cost to the society.^[1]^ It has been reported that its prevalence among children and teenagers was as high as 50% in Taiwan,^[2]^ 67.3% in Chinese mainland,^[3]^ 70% in Singapore,^[4]^ and even 96.5% in Korea.^[5]^ It seriously affects the quality of vision of children and teenagers. Thus, there is an urgent need to develop effective treatment strategies for myopia.

Myopia is an ocular disease characterized by an abnormally elongated eyeball, which cannot be rescued by optical lenses or refractive surgeries. To date, laser in situ keratomileusis (LASIK) has been the standard refractive surgery for treating myopia owing to its safety and efficacy.^[6]^ However, conventional LASIK has the potential increase in corneal higher order aberrations (HOA) caused by an oblate central corneal surface, which may cause postoperative halos, glare, and night vision difficulties. With the development of surgical instruments, the technique has gradually evolved accordingly. A better refractive outcome for improving vision quality has gradually being explored in clinical research. Recently, *Q*-value-guided LASIK is regarded as a relatively novel surgical option. It provides wavefront-guided corneal aspheric ablation to maintain preoperative and postoperative corneal shape, as evaluated by the *Q*-value. This device may be a promising tool to provide benefits in vision quality. Compared with conventional LASIK procedure, *Q*-value-guided LASIK also allows the surgeon to reduce the amount of tissue removal by approximately 30%.^[7,8]^ However, there were conflicting reports about the postoperative visual recovery and corneal stability of *Q*-value-guided LASIK. Meta-analysis can get a relatively precise and accurate estimation through incorporating all available data using statistical tool. Thus, the meta-analysis was to explore the safety and efficacy of *Q*-value-guided LASIK for treating myopia.

## 2 Materials and methods

### 2.1 Ethics statement

The Preferred Reporting Items for Systematic Reviews and Meta-Analysis guidelines was used to perform the current meta-analysis.^[9]^ No patient's privacy or clinical sample was involved in this study, hence the ethical approval was not required.

### 2.2 Identification and eligibility of relevant studies

Literature resources including PubMed, Cochrane Library, Embase, China Biology Medicine disc and China National Knowledge Infrastructure were searched for eligible literatures. The search terms were composed of myopia (eg, myopia, short-sight and nearsighted), LASIK (eg, LASIK and Keratomileusis, Laser In Situ). Last search of current investigation was updated on November 21, 2018. The language was limited to English and Chinese. We identified other relevant articles according to scan all retrieved articles and reviews. We treated them independently if the different groups were found in a reported article.

### 2.3 Inclusion and exclusion criteria

Studies followed the 2 criteria could be identified:

(1) all randomized controlled trials (RCTs) and cohorts;
(2) The studies provided available data;

As per the exclusion criteria:

(1) the available data was absent;
(2) similar or duplicate study (When the same or similar cohort was applied, the most complete information was included);
(3) other types of articles including reviews or abstracts.

### 2.4 Data extraction

In the light of inclusion and exclusion criteria, we extracted the relevant information from each eligible publication. If disagreements were noticed, we are clearly open to discussion by each other (Zhang Kaiping and Fang Xiang), or reviewed by a third author (Chao Min). The information on first author, publication year, study country, follow-up, laser Instrument, the number of patients and eyes, age, preoperative spherical equivalent (SE) and study design was collected by 2 authors independently. We did not contact any authors of the original researches even though the essential information could not be available. Besides, country was divided into China and others. Number of eyes enrolled included ≧100 and < 100. Study design was stratified into 2 groups: RCT and cohort. The Newcastle-Ottawa Scale consisted of selection, comparability of the groups and ascertainment of exposure was introduced to evaluate the included publication's quality. The Newcastle-Ottawa Scale scores were 0 to 10 stars. If 1 included study obtained no less than 7 stars, it could be regarded as high-quality.^[10]^

### 2.5 Outcome measures

The outcome measures included the number of postoperative uncorrected visual acuity (UCVA) of 20/20 or better, postoperative UCVA, preoperative and postoperative *Q*-value, postoperative refractive SE, the number of postoperative SE within ±0.5 D, HOA, coma-like aberration and spherical-like aberration.

### 2.6 Statistical analysis

RevMan software (version 5.3; Cochrane Collaboration, Oxford, United Kingdom) and STATA (version 12.0; Stata Corporation, College Station, Texas) were introduced to analyze the data in current meta-analysis. Odds ratio (ORs) with 95% confidence interval (CIs) were calculated for the dichotomous outcomes. For the continuous measures, mean difference (MDs) with 95% CIs were used, and a *P* < .05 was considered to be statistically significant difference. The heterogeneity has been assessed via chi-square-based *Q* and extent of inconsistency (*I*^2^) test across studies (no heterogeneity *I*^2^ < 25%, moderate heterogeneity *I*^2^ = 25%-50%, extreme heterogeneity *I*^2^ > 50%).^[11]^ In case of extreme heterogeneity (I^2^ > 50% or *P* < .01 for *Q* test), we used random-effects (DerSimonian and Laird method) model.^[12]^ Otherwise, fixed-effects (Mantel-Haenszel method) model was introduced.^[13]^

Subgroup analyses were performed on study design (RCTs versus cohorts), country (China versus Others) and number of eyes enrolled (≥100 versus < 100). Additionally, 1-way sensitivity analyses individually removed publications in meta-analysis were conducted to assess results’ stability. Publication bias was estimated using Begg and Egger tests.^[14]^

## 3 Results

### 3.1 Characteristics of Eligible Studies

**Figure 1:**
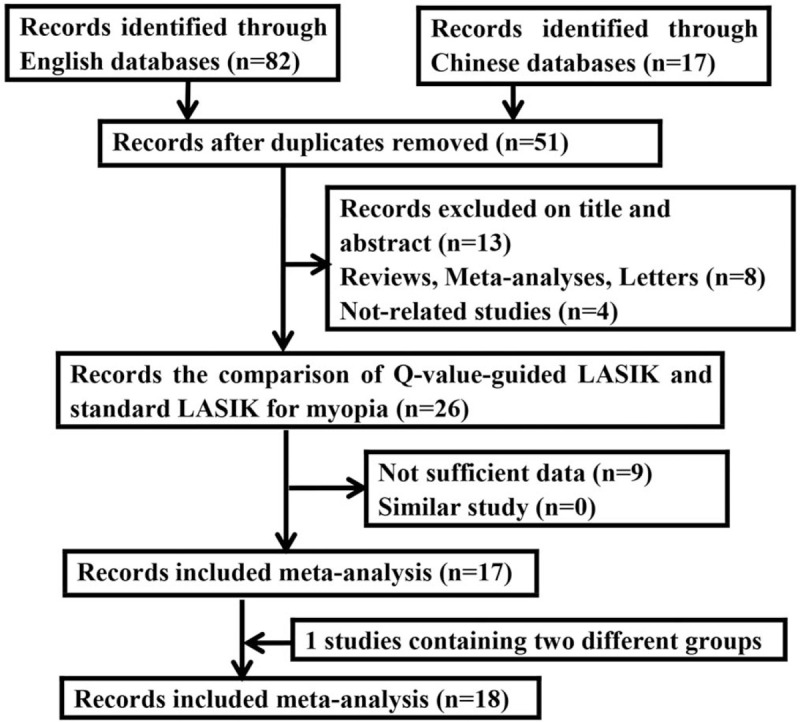
Flow diagram of the study selection process in the meta-analysis.

A total of 17 studies with 2640 patients and 3358 eyes satisfied the eligible studies.^[15–31]^ Among them, Villa C et al study investigated 2 different case-control studies and we separated them independently into meta-analysis.^[20]^ Therefore, the current meta-analysis was established based on 18 studies (Fig. 1). Of these studies, 6 RCTs and twelve cohorts were included. The number of eyes ranged from 48 to 755. The main characteristics of the included studies were shown in Table 1.

**Table 1:** Characteristics of studies included in the meta-analysis.

					*Q*-adjusted LASIK			Standard LASIK				
								
Authors	Year	Country	Follow-up (mo)	Laser Instrument	Eyes/Patients(n)	Age (yrs)	Preoperative SE (D)	Eyes/Patients (n)	Age (yrs)	Preoperative SE (D)	Design	NOS
Li et al^[15]^	2012	China	3	Allegretto Wave Eye-Q 400 Hz (Wavelight AG, Germany)	32/32	21 ± 2.50	DS: -4.38 ± -0.58 DC: -0.82 ± -0.25	16/16	23 ± 3.1	DS: -3.39 ± -0.82 DC: -0.61 ± -0.46	Cohort	6
Zheng et a*l*^[16]^	2011	China	>6	Technolas 217-z100 excimer laser (Bausch and Lomb)	132/66	18–43	−2.25	100/50	18–39	−2	Cohort	7
Zhou et al^[17]^	2010	China	1	Allegretto Wave Eye-Q 400 Hz (Wavelight AG)	27/27	22.6 (18–35)	−4.56 ± 1.69	27/27	22.6 (18–35)	−4.38 ± 1.80	RCT	7
Xin et al^[18]^	2010	China	36	Technolas 217-z100 excimer laser (Bausch and Lomb)	367/189	NA	NA	194/100	NA	NA	RCT	6
Igarashi et al^*[19*]^	2009	Japan	3	Technolas 217-z100 excimer laser (Bausch and Lomb)	28/15	36.4 ± 5.8	−5.13 ± 1.23	33/18	32.9 ± 8.3	−5.63 ± 0.88	Cohort	6
Villa et al^[20]^	2009	Spain	3	Allegretto Wave Eye-Q 400 Hz (Wavelight AG, Germany)	48/24 ^a^ 40/40^b^	32.3^a^ 31.3^b^	−3.4^a^ –4^b^	76/38^a^ 40/40^b^	35.2^a^ 31.3^b^	−3.7^a^ −4^b^	Cohort	8
Liu et al^[21]^	2008	China	>12	Astrascan XL 200 Hz (LaserSigh)	106/53	27.9 ± 4.86	−6.57 ± 1.81	102/51	25.4 ± 5.85	−5.99 ± 2.53	RCT	7
Wei et al^[22]^	2008	China	1	NA	276/139	20.49 ± 3.31	−4.52 ± 1.77	479/241	22.79 ± 4.42	−4.55 ± 1.91	Cohort	7
Ma et al^[23]^	2008	China	>6	Allegretto Wave Eye-Q 400 Hz (Wavelight AG, Germany)	86/43	NA	NA	86/43	NA	NA	Cohort	8
Zou et al^[24]^	2008	China	3	Technolas 217-z100 excimer laser (Bausch and Lomb)	152/80	26.56 ± 4.97	DS: −5.79 ± −2.18 DC: −0.79 ± −0.41	181/100	25.4 ± 5.17	DS: −5.21 ± −1.41 DC: −0.60 ± −0.49	RCT	8
Xu et al^[25]^	2008	China	3	Allegretto Wave Eye-Q 400 Hz (Wavelight AG, Germany)	46/23	25.5 ± 6.02	−5.48 ± 2.38	44/22	24.7 ± 5.86	−5.62 ± 2.63	Cohort	7
Zhou et al^[26]^	2008	China	6	Mel 80 (Carl Zeis, Germany)	38/38	27.1 ± 4.8	−3.46 ± 1.62	41/41	26.4 ± 6.1	−3.59 ± 1.68	Cohort	6
Cai et al^[27]^	2008	China	1	Astrascan XL 200 Hz (LaserSigh)	64/32	24.3 ± 7.2	DS: −3.24 ± 1.21 DC: −0.46 ± 0.29	64/32	25.1 ± 6.7	DS: −3.13 ± 1.09 DC: −0.58 ± 0.31	RCT	6
Huang et al^[28]^	2008	China	3	Technolas 217-z100 excimer laser (Bausch and Lomb)	43/43	23 ± 4	-4.83 ± 1.28	41/41	25 ± 4	−5.01 ± 1.65	RCT	7
Chen et al^[29]^	2007	China	6	Astrascan XL 200 Hz (LaserSigh)	66/33	24.61 ± 5.92	−5.18 ± 1.62	59/30	24.2 ± 6.46	−5.26 ± 1.65	Cohort	8
Liu et al^[30]^	2007	China	1	Technolas 217-z100 excimer laser (Bausch and Lomb)	51/28	24.65 ± 0.91	DS: −6.71 ± 0.91 DC: −0.68 ± 0.09	51/26	23.87 ± 1.05	DS: −6.62 ± 0.21 DC: −0.64 ± 0.07	Cohort	8
Shen et al^[31]^	2005	China	6	Allegretto Wave Eye-Q 400 Hz (Wavelight AG, Germany)	64/32	NA	−6.22 ± 2.22	58/29	NA	−6.19 ± 2.17	Cohort	6

### 3.2 Meta-analysis results

#### 3.2.1 Postoperative UCVA of 20/20 or Better

**Figure 2:**
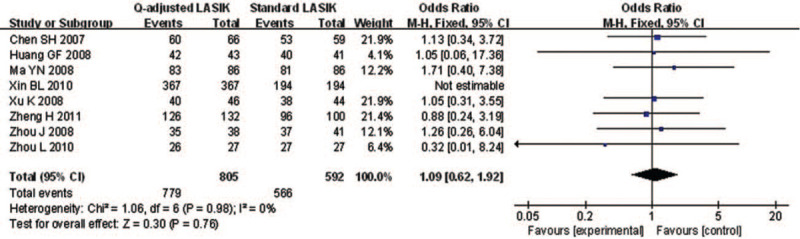
Forest plot of postoperative UCVA of 20/20 or better between Q-value-guided LASIK and standard LASIK for myopia. LASIK = Laser in situ keratomileusis, UCVA = uncorrected visual acuity.

Eight studies reported the postoperative UCVA of 20/20 or better of *Q*-value-guided LASIK and standard LASIK for myopia. No heterogeneity was found (*I*^2^ = 0.0%), so fixed effects model was used to calculate the combined OR and 95% CI. As a result, the pooled result revealed that no significant difference was detected between the 2 paired groups (OR = 1.09; 95% CI: 0.62, 1.92; *P* = .763) (Fig. 2)

#### 3.2.2 Postoperative UCVA.

**Figure 3:**
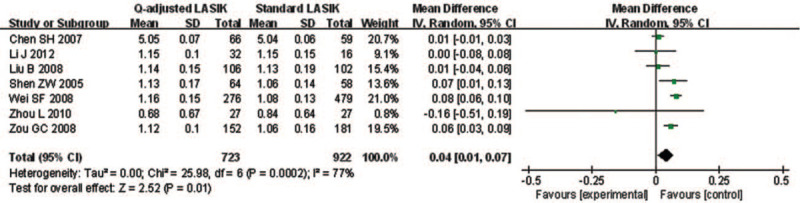
Forest plot of postoperative UCVA between Q-value-guided LASIK and standard LASIK for myopia. LASIK = Laser in situ keratomileusis, UCVA = uncorrected visual acuity.

Seven studies compared the postoperative UCVA between *Q*-value-guided LASIK and standard LASIK for myopia. Apparent heterogeneity was found (*I*^2^ = 76.9%), so random effects model was applied to calculate MD (95% CI). A statistically significant difference was found in postoperative UCVA (MD = 0.04; 95% CI: 0.01, 0.07; *P* = .012) (Fig. 3).

#### 3.2.3 Preoperative and postoperative *Q*-value

**Figure 4:**
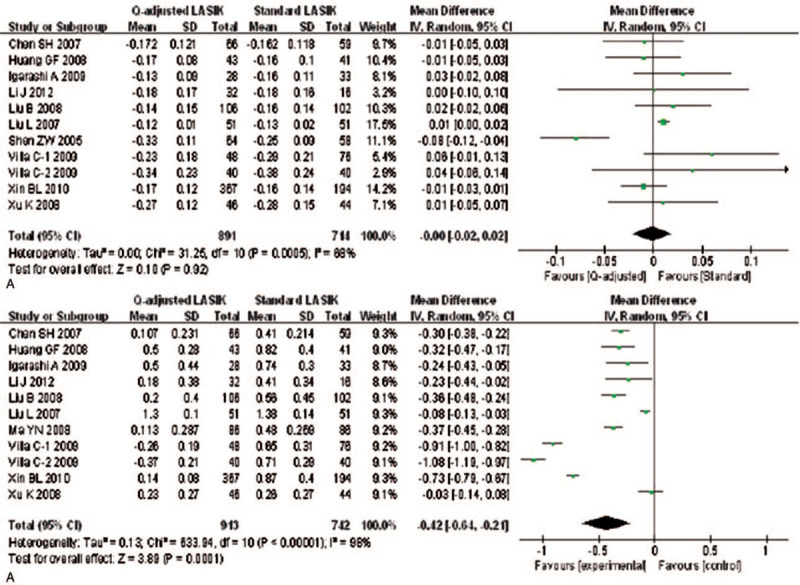
Forest plot of preoperative and postoperative Q-value between Q-value-guided LASIK and standard LASIK for myopia. A: preoperative Q-value; B: postoperative Q-value. LASIK = Laser in situ keratomileusis.

There are eleven studies to detect the preoperative and postoperative *Q*-value between 2 paired groups. An evident heterogeneity was detected among the study results (*I*^2^ = 68% and 98.4%), so random effects model was applied to calculate the combined MD and 95% CI. No significant differences were found in preoperative *Q*-value (MD = -0.00; 95% CI: -0.02, 0.02; *P* = .922) (Fig. 4A). However, there was a statistically significant difference in postoperative *Q* values between 2 paired groups (MD = -0.42; 95% CI: -0.64, -0.21; *P* < .001) (Fig. 4B).

#### 3.2.4 Postoperative refractive SE

**Figure 5:**
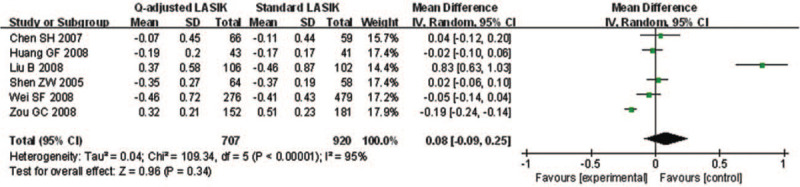
Forest plot of postoperative refractive SE between Q-value-guided LASIK and standard LASIK for myopia. LASIK = Laser in situ keratomileusis, SE = spherical equivalent.

Only 6 studies explored postoperative refractive SE of *Q*-value-guided LASIK and standard LASIK. Apparent heterogeneity was found (*I*^2^ = 95.4%), so random effects model was applied to calculate the combined MD and 95% CI. There was no significant difference between 2 paired groups (MD = 0.08; 95% CI: -0.09, 0.25; *P* = .336) (Fig. 5).

#### 3.2.5 Postoperative SE within ±0.5 D of Target Refraction

**Figure 6:**
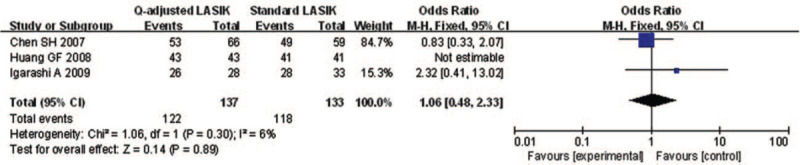
Forest plot of postoperative SE within ±0.5 D of target refraction between Q-value-guided LASIK and standard LASIK for myopia. LASIK = Laser in situ keratomileusis, SE = spherical equivalent.

Only 3 studies were involved to explore the number of postoperative SE within ±0.5 D. No heterogeneity was found (*I*^2^ = 0.0%), so fixed effects model was used to calculate the combined OR and 95% CI. The forest plot showed that no significant difference was found in postoperative SE within ±0.5 D (OR = 1.06; 95% CI: 0.48, 2.33; *P* = .886) (Fig. 6).

#### 3.2.6 Postoperative aberration

**Figure 7:**
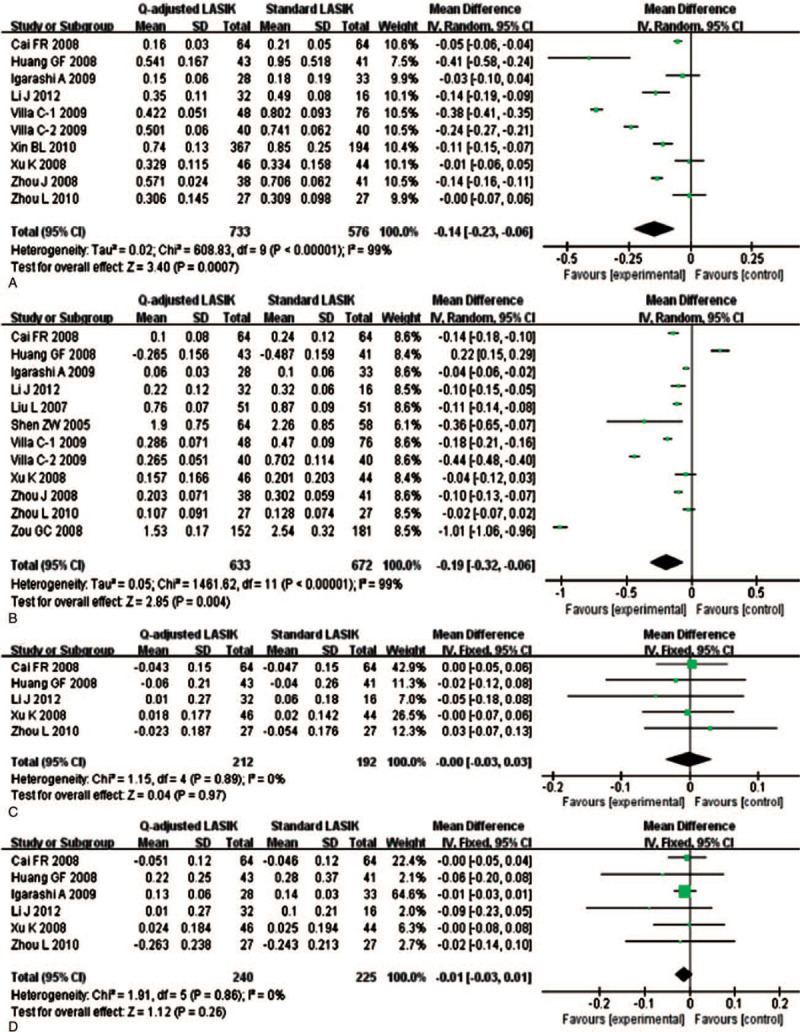
Forest plot of different postoperative aberration between Q-value-guided LASIK and standard LASIK for myopia. A: HOA; B: spherical aberrations; C: horizontal coma-like aberration; D: vertical coma-like aberration. LASIK = Laser in situ keratomileusis.

Postoperative aberration included HOA, coma-like aberration and spherical-like aberration. Among them, coma-like aberration contained horizontal and vertical coma-like aberration. Ten and twelve studies explored HOA and spherical-like aberration, respectively. Apparent heterogeneity was found (*I*^2^ = 98.5% and 99.2%), so random effects model was applied to calculate the combined MD and 95% CI. Compared to the standard LASIK group, HOA (MD = -0.14; 95% CI: -0.23, -0.06; *P* = .001) (Fig. 7A) and spherical aberrations (MD = -0.19; 95% CI: -0.32, -0.06; *P* = .004) (Fig. 7B) increased more in the *Q*-value-guided LASIK group, and there were statistically differences. Additionally, there were 5 and 6 studies involved in horizontal and vertical coma-like aberration, respectively. No heterogeneity was found (*I*^2^ = 0.0% and 0.0%). Consequently, no significant differences were found in coma-like aberration between 2 paired groups (horizontal: MD = -0.00; 95% CI: -0.03, 0.03; *P* = .966; vertical: MD = -0.01; 95% CI: -0.03, 0.01; *P* = .263) (Fig. 7C-7D).

#### 3.2.7 Subgroup-analysis results

Subgroup analyses were performed according to the study design (RCTs versus cohorts), country (China versus Others) and number of eyes enrolled (≧100 versus < 100). As shown in Table 2, significant statistically difference were found in sub-analyses regarding postoperative *Q*-value (RCTs: MD = -0.48; 95% CI: -0.77, -0.18; *P* = .002; cohort: MD = -0.41; 95% CI: -0.68, -0.14; *P* = .003), HOA (RCTs: MD = -0.10; 95% CI: -0.17, -0.03; *P* = .006; cohort: MD = -.16; 95% CI: -0.26, -0.05; *P* = .004) and spherical-like aberration (cohort: MD = -0.16; 95% CI: -0.25, -0.07; *P* = .001). As for subgroup of country, similar results were found in postoperative *Q*-value (country: MD = -0.30; 95% CI: -0.51, -0.10; *P* = .003; other: MD = -0.75; 95% CI: -1.12, -0.38; *P* = .000), HOA (country: MD = -0.10; 95% CI: -0.14, -0.05; *P* = .000; other: MD = -0.22; 95% CI: -0.37, -0.07; *P* = .004) and spherical-like aberration (other: MD = -0.22; 95% CI: -0.43, -0.01; *P* = .039) (Table 3). Likewise, we also detected similar results via subgroup analysis on study eye sizes. Postoperative UCVA (≧100: MD = 0.05; 95% CI: 0.01, 0.08; *P* = .006), postoperative *Q*-value (≧100: MD = -0.46; 95% CI: -0.73, -0.18; *P* = .001), HOA (<100: MD = -0.12; 95% CI: -0.20, -0.05; *P* = .001) and spherical-like aberration (≧100: MD = -0.36; 95% CI: -0.64, -0.08; *P* = .011) in *Q*-value-guided LASIK group exhibited statistically significant differences compared to control (Table 4).

**Table 2:** Subgroup analyses on study design.

Study design (RCTs versus Cohorts)	Studies	Eyes	OR or MD (95%CI)	*P*	I^2^
Postoperative UCVA of 20/20 or better	8	1397	OR 1.09 (0.62, 1.92)	.763	0.0%
RCT	3	699	OR 0.61 (0.08, 4.70)	.633	0.0%
Cohort	5	698	OR 1.15 (0.64, 2.06)	.647	0.0%
Postoperative UCVA	7	1645	**MD 0.04 (0.01, 0.07)**	.012	76.9%
RCT	3	595	MD 0.03 (−0.02, 0.08)	.180	56.2%
Cohort	4	1050	MD 0.04 (−0.00, 0.09)	.070	86.0%
Preoperative Q-value	11	1605	MD −0.00 (−0.02, 0.02)	.922	68.0%
RCT	3	853	MD −0.00 (−0.02, 0.01)	.668	0.0%
Cohort	8	752	MD 0.00 (−0.03, 0.03)	.935	75.0%
Postoperative Q-value	11	1655	**MD** −**0.42 (**−**0.64,** −**0.21)**	.000	98.4%
RCT	3	853	MD −0.48 (−0.77, −0.18)	.002	96.0%
Cohort	8	802	MD −0.41 (−0.68, −0.14)	.003	98.5%
Postoperative refractive SE	6	1627	MD 0.08 (−0.09, 0.25)	.336	95.4%
RCT	3	1002	MD 0.19 (−0.17, 0.54)	.299	98.0%
Cohort	3	625	MD −0.00 (−0.06, 0.05)	.897	0.0%
HOA	10	1309	**MD** −**0.14 (**−**0.23,** −**0.06)**	.001	98.5%
RCT	4	827	MD −0.10 (−0.17, −0.03)	.006	89.4%
Cohort	6	482	MD −0.16 (−0.26, −0.05)	.004	98.4%
Horizontal coma-like aberration	5	404	MD −0.00 (−0.03, 0.03)	.966	0.0%
RCT	3	266	MD 0.00 (−0.04, 0.05)	.816	0.0%
Cohort	2	138	MD −0.01 (−0.07, 0.05)	.688	0.0%
Vertical coma-like aberration	6	455	MD −0.01 (−0.03, 0.01)	.263	0.0%
RCT	3	266	MD −0.01 (−0.05, 0.03)	.577	0.0%
Cohort	3	189	MD −0.01 (−0.03, 0.01)	.331	0.0%
Spherical-like aberration	12	1305	**MD** −**0.19 (**−**0.32,** −**0.06)**	.004	99.2%
RCT	4	599	MD −0.24 (−0.69, 0.22)	.308	99.7%
Cohort	8	706	MD −0.16 (−0.25, −0.07)	.001	97.9%

**Table 3:** Subgroup analyses on country.

Country (China versus Others)	Studies	Eyes	OR or MD (95%CI)	*P*	*I*^2^
Preoperative *Q*-value	11	1605	MD −0.00 (−0.02, 0.02)	.922	68.0%
China	8	1340	MD −0.01 (−0.03, 0.01)	.398	74.7%
Others	3	265	MD 0.04 (0.00, 0.08)	.019	0.0%
Postoperative *Q*-value	11	1655	MD −0.42 (−0.64, −0.21)	.000	98.4%
China	8	1390	MD −0.30 (−0.51, −0.10)	.003	97.9%
Others	3	265	MD −0.75 (−1.12, −0.38)	.000	96.4%
HOA	10	1309	MD −0.14 (−0.23, −0.06)	.001	98.5%
China	7	1044	MD −0.10 (−0.14, −0.05)	.000	92.4%
Others	3	265	MD −0.22 (−0.37, −0.07)	.004	98.3%
Vertical coma-like aberration	6	455	MD −0.01 (−0.03, 0.01)	.263	0.0%
China	5	404	MD −0.01 (−0.05, 0.02)	.424	0.0%
Others	1	51	MD −0.01 (−0.03, 0.01)	.423	/
Spherical-like aberration	12	1305	MD −0.19 (−0.32, −0.06)	.004	99.2%
China	9	1040	MD −0.18 (−0.37, 0.00)	.055	99.3%
Others	3	265	MD −0.22 (−0.43, −0.01)	.039	99.3%

**Table 4:** Subgroup analyses on study eye sizes.

Eye sizes (≧100 versus < 100)	Studies	Eyes	OR or MD (95%CI)	*P*	*I*^2^
Postoperative UCVA of 20/20 or better	8	1397	OR 1.09 (0.62, 1.92)	.763	0.0%
≧100	4	1090	OR 1.16 (0.55, 2.44)	.697	0.0%
<100	4	307	OR 1.00 (0.42, 2.38)	.991	0.0%
Postoperative UCVA	7	1645	MD 0.04 (0.01, 0.07)	.012	76.9%
≧100	5	1543	MD 0.05 (0.01, 0.08)	.006	82.8%
<100	2	102	MD −0.01 (−0.09, 0.07)	.839	0.0%
Preoperative Q-value	11	1605	MD −0.00 (−0.02, 0.02)	.922	68.0%
≧100	6	1242	MD −0.01 (−0.03, 0.02)	.678	82.9%
<100	5	363	MD 0.01 (−0.02, 0.03)	.547	0.0%
Postoperative Q-value	11	1655	MD −0.42 (−0.64, −0.21)	.000	98.4%
≧100	6	1292	MD −0.46 (−0.73, −0.18)	.001	98.9%
<100	5	363	MD −0.38 (−0.83, 0.06)	.093	97.9%
Postoperative refractive SE	6	1627	MD 0.08 (−0.09, 0.25)	.336	95.4%
≧100	5	1543	MD 0.11 (−0.11, 0.33)	.315	96.3%
<100	1	84	MD −0.02 (−0.10, 0.06)	.621	/
HOA	10	1309	MD −0.14 (−0.23, −0.06)	.001	98.5%
≧100	3	813	MD −0.18 (−0.40, 0.04)	.109	99.6%
<100	7	496	MD −0.12 (−0.20, −0.05)	.001	94.6%
Horizontal coma-like aberration	5	404	MD −0.00 (−0.03, 0.03)	.966	0.0%
≧100	1	128	MD 0.00 (−0.05, 0.06)	.880	/
<100	4	276	MD −0.00 (−0.05, 0.04)	.851	0.0%
Vertical coma-like aberration	6	455	MD −0.01 (−0.03, 0.01)	.263	0.0%
≧100	1	128	MD −0.00 (−0.05, 0.04)	.814	/
<100	5	327	MD −0.01 (−0.04, 0.01)	.253	0.0%
Spherical-like aberration	12	1305	MD −0.19 (−0.32, −0.06)	.004	99.2%
≧100	5	809	MD −0.36 (−0.64, −0.08)	.011	99.6%
<100	7	496	MD −0.08 (−0.20, 0.05)	.238	98.6%

### 3.3 Sensitivity analysis and publication bias

Each study here was deleted at a time to assess the specific effect of the individual data on the pooled results, and one-way sensitivity analysis suggested the results were relatively stable. The Begg test (*P* = .06 to 1.000) and Egger test (*P* = .021 to .735) were applied to all of the outcome measures. No publication bias was found in all outcome measures rather than postoperative refractive SE (Egger: *P* = .021).

## 4 Discussion

Q-value-guided LASIK is new technology now approved for clinical use. However, there were controversial reports about its postoperative visual recovery and corneal stability. Meta-analysis could get a relatively precise estimation from different inconsistent studies. We tried to explore its safety and efficacy in current research. As a result, *Q*-value-guided LASIK is a safe, effective and predictable surgical options for treating myopia. Meanwhile, Q-value-guided LASIK shows obvious superiority in postoperative *Q*-value, HOA and spherical-like aberration. It could provide benefits for improvement of vision quality, and have a relatively smaller increase in the differential postoperative *Q*-value after surgery. Generally, the outer surface of the human cornea is physiologically conical rather than a sphere. A significant variation of physiologic asphericity is shown ranging from mild oblate to moderate prolate.^[32]^ Therefore, it is necessary to introduce a shape factor to characterize the amount of asphericity of the cornea numerically, the so-called *Q*-factor. The *Q* value is negative for most eyes and not related to the degree of myopia.^[33,34]^*Q* value mathematically reflects corneal asphericity, which can be defined to variations in radius of curvature from apex to periphery. ^[35,36]^

To date, LASIK is a common corneal surgery for myopia and astigmatism.^[37]^ It makes the cornea undergo a anatomical change, from its initially prolate shape (*Q* < 0) with a steeper central area and flat peripheral area to an oblate shape (*Q* > 0) with a flat center and steep periphery.^[38–40]^ LASIK can reduce refractive error and improve uncorrected visual acuity, but several problems still must be resolved regarding postoperative visual function and contrast sensitivity.^[41]^ Increased higher-order optical aberrations after laser refractive surgery was found to be a potentially major factor in visual quality.^[42]^ A further study is required to determine the exact *Q*-values after surgery. With the development and maturation of refractive surgery, relatively high surgical efficacy based on Q-value has been introduced for treating myopia. *Q*-value guided surgery aims to minimize changes of the corneal anterior surface asphericity in order to reduce corneal ablation depth, which impacts mostly on visual quality.^[43–45]^ However, there were conflicting reports about the postoperative visual recovery and corneal stability of Q-value-guided LASIK. Thus, to explore its safety and efficacy, we performed the current meta-analysis to compare *Q*-value-guided LASIK with standard LASIK.

Due to significant heterogeneity of the current meta-analysis, careful interpretation and search for influencing factors were required. LASIK for the correction of myopia is primarily concerned with production of refractive changes by corneal flattening in relation to the amount of refractive error. The Technolas was gradually approved for clinical use in China. It features a new algorithm with preoperative assessment of the Q-value together with subjective refraction. In presented research, the included studies mainly focused on Chinese and English literatures, which may influence the ultimate results. Additionally, differences in the study design should be considered as potential sources of heterogeneity. Sample size also have an impact on heterogeneity. The differences in the baseline, such as age or gender, are likely to be significant factors contributing to the results.

Actually, there are several important limitations. Firstly, only published studies may not provide sufficient evidences. Secondly, only English and Chinese literatures were explored, which may influence the ultimate results. Meanwhile, the extreme heterogeneity suggested there are potential or undiscovered factors. The impact of those factors could not be formally explored through subgroup analysis. Whereas, in spite of aforementioned limitations, it also has been proven that Q-value-guided LASIK is a safe, effective and predictable surgical options for treating myopia.

## Author contributions

**Conceptualization:** Kai-Ping Zhang.

**Data curation:** Kai-Ping Zhang.

**Formal analysis:** Kai-Ping Zhang, Xiang Fang.

**Investigation:** Xiang Fang, Yin Zhang.

**Methodology:** Xiang Fang, Yin Zhang.

**Project administration:** Kai-Ping Zhang, Min Chao.

**Resources:** Kai-Ping Zhang.

**Software:** Kai-Ping Zhang,

**Supervision:** Kai-Ping Zhang, Min Chao.

**Validation:** Kai-Ping Zhang, Yin Zhang, Min Chao.

**Visualization:** Min Chao.

**Writing – original draft:** Yin Zhang, Min Chao.

**Writing – review & editing:** Kai-Ping Zhang, Xiang Fang, Yin Zhang, Min Chao.
